# Lack of FADD in Tie-2 expressing cells causes RIPK3-mediated embryonic lethality

**DOI:** 10.1038/cddis.2016.251

**Published:** 2016-09-01

**Authors:** Cunxian Fan, Wenjuan Pu, Xiaoxia Wu, Xixi Zhang, Lingjuan He, Bin Zhou, Haibing Zhang

**Affiliations:** 1 Key Laboratory of Nutrition and Metabolism, Institute for Nutritional Sciences, Shanghai Institutes for Biological Sciences, Chinese Academy of Sciences, University of Chinese Academy of Sciences, Shanghai 200031, China

*Dear Editor,*

Programmed cell death has an essential role in development and homeostasis of mammalians. Fas-associated death domain (FADD) interacts with the death domain of receptors, leading to the activation of caspase-8, which subsequently activates several downstream caspases and finally executes apoptosis.^1^ Ablation of *Caspase-8* or *Fadd* resulted in embryonic lethality at around E10.5, which implicates a non-apoptosis function of these proteins in embryonic development.^2^ Recently, extensively genetic studies have shown that embryonic lethality caused by *Fadd* (or *Caspase-8*) deletion can be rescued by *Ripk3* or *Ripk1* ablation.^2^ However, it remains unclear which targeted cell type is responsible for the lethality of *Fadd*^−/−^ mice.

Mice with conditional deletion of *Fadd* in immune cells, skin or intestine produced no lethality.^2^ Given the fact that mice with *Caspase-8* deficiency in endothelium, employing Tie1-cre promotor, resemble the embryonic lethality of *Caspase-8* germline knockout associated with cardiac defects,^3^ we hypothesized that embryonic lethality of *Fadd* knockout might also attribute to the loss of *Fadd* in endothelial cells. To directly test this hypothesis, we took advantage of the mice that expressed a functional FADD:GFP fusion gene to reconstitute *Fadd*^−/−^ mice, and generated tissue-specific *Fadd* deletion mice using cre-recombinase under the control of tissue-specific promoter, which were reported previously.^4^

First, we specifically deleted FADD:GFP in cardiomyocytes and cardiac progenitor cells by crossing the mice (*Fadd*^−/−^*Fadd:gfp*^+^) individually with transgenic mice expressing the *cTnt-cre* and *Nkx2.5-cre*. *cTnt-Cre* efficiently delete target genes in myocardium and *Nkx2.5-cre* targets cardiomyocyte progenitors.^5, 6^ We found that both *Fadd*^−/−^*Fadd:gfp*^+^*cTnt-cre*^+^ and *Fadd*^−/−^*Fadd:gfp*^+^*Nkx2.5-cre*^+^ mice develop normally at E11.5 (Figure 1a). These data indicate that it is loss of *Fadd* in other types of cells causing embryonic death of *Fadd*^−/−^ mice, not cardiomyocytes or cardiac progenitor cells. We then generated *Fadd* deficiency in Tie-2 expressing cells by crossing *Fadd*^−/−^*Fadd:gfp*^+^ mice with a transgenic line that expresses cre-recombinase under the control of Tie-2 promoter. In contrast, *Fadd*^−/−^*Fadd:gfp*^+^*Tie2-cre*^+^ mice died at E11.5 with the same cardiovascular defects as *Fadd*^−/−^ mice, such as vessel defect and pericardial bleeding, suggesting that hemodynamic failure resulting in embryonic death could be owing to abnormal cardiovascular development (Figure 1a). Whole-mount staining for endothelial cell marker PECAM and FADD surrogates GFP showed that FADD:GFP was expressed in endothelial cells in *Fadd*^−/−^*Fadd:gfp*^+^ mice (yellow). However, FADD:GFP was not detected in endothelial cells of *Fadd*^+/−^*Fadd:gfp*^+^*Tie2-cre*^+^ embryo (Figure 1b), whereas FADD:GFP was still expressed in non-endothelial cells such as cardiomyocytes (Figure 1b). These data indicate that Tie2-cre efficiently ablates FADD:GFP in endothelial cells. Compared with normal embryos, *Fadd*^−/−^*Fadd:gfp*^+^*Tie2-cre*^+^ displayed a low degree of trabeculation in the walls of the common ventricular chamber and endocardial cushion defect by reduced endothelial-to-mesenchymal formation (Figure 1c), suggesting that loss of *Fadd* in endothelial cells causes endocardium-related cardiac development defect. Although the lethality of *Fadd*^−/−^*Fadd:gfp*^+^*Tie2-cre*^+^ is caused by the same pathology as *Fadd*^−/−^ embryo at E11.5,^7, 8^ we asked whether this lethality of *Fadd*^−/−^*Fadd:gfp*^+^*Tie2-cre*^+^ is mediated by RIPK3 as *Fadd*^−/−^ mice. Therefore, we crossed *Ripk3* knockout allele to the *Fadd*^−/−^*Fadd:gfp*^+^*Tie2-cre*^+^ mice and found that embryonic lethality of *Fadd*^−/−^*Fadd:gfp*^+^*Tie2-cre*^+^ mice at E11.5 was rescued by *Ripk3* deletion. Furthermore, *Fadd*^−/−^*Fadd:gfp*^+^*Tie2-cre*^+^*Ripk3*^−/−^ embryos displayed normal degree of trabeculation in the walls of the common ventricular chamber and normal cushion development as *Fadd*^−/−^*Fadd:gfp*^+^ embryos (Figure 1c). Given that Tie2 is predominantly expressed in endothelial cells and hematopoietic cells, embryonic lethality of *Fadd*^−/−^*Fadd:gfp*^+^*Tie2-cre*^+^ mice at E11.5 might be owing to *Fadd* deletion in both endothelial and hematopoietic populations. In addition, hematopoietic stem cells are mainly derived from aortic endothelial cells during early embryonic development, and the role of FADD in hematopoietic development could also be secondary to the defect in endothelial cells. More specific genetic tools that could distinguish hematopoietic cells and endothelial cells are needed to dissect the roles of FADD in these two populations. Taken together, these results demonstrated that RIPK3-mediated signaling in Tie-2 expressing cells was responsible for the embryonic lethality of *Fadd*^−/−^ with cardiac failure. Further, mechanistic study of cell death in these cell populations will be important for understanding the function of cell death during embryonic development.

## Figures and Tables

**Figure 1:**
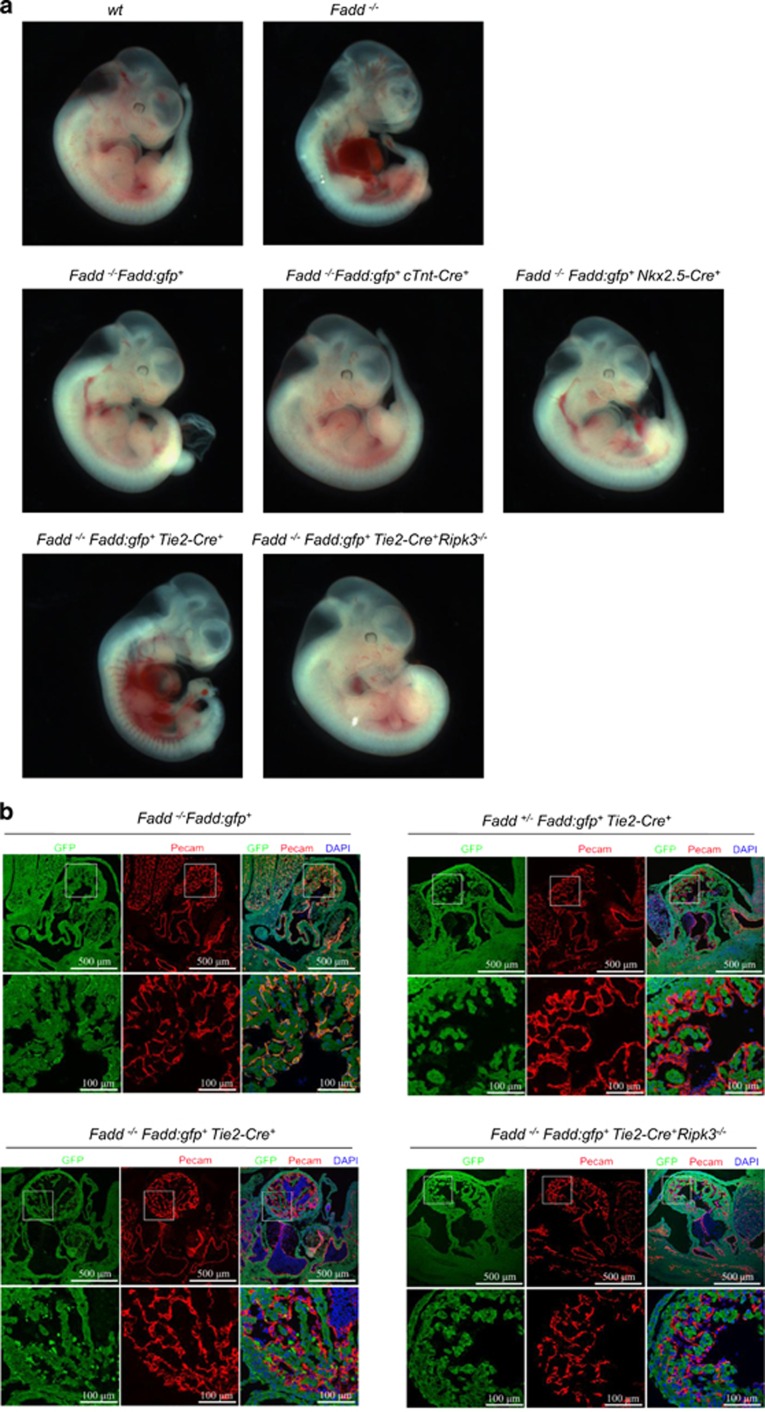

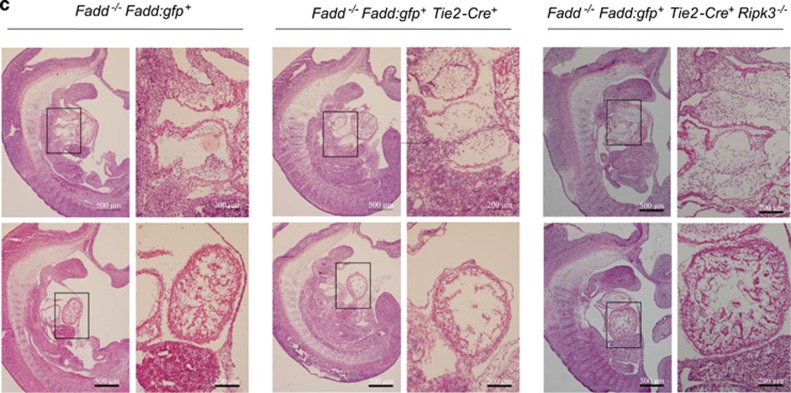
Loss of FADD in Tie-2 expressing cells leads to embryonic lethality at E11.5 with heart defects, which can be rescued by RIPK3 knockout. (**a**) Whole-mount images of E11.5 embryos of the indicated genotypes. Compared with wild-type embryos, *Fadd*^−/−^*Fadd:gfp*^+^*cTnt-cre*^+^ and *Fadd*^−/−^*Fadd:gfp*^+^*Nkx2.5-cre*^+^ embryos were normal. *Fadd*^−/−^*Fadd:gfp*^+^*Tie2-Cre*^+^ embryos showed cardiovascular defects that included bleeding and pericardial edema, and no such defects were observed in *Fadd*^−/−^*and Fadd*^−/−^*Fadd:gfp*^+^*Tie2-Cre*^+^*Ripk3*^−/−^ embryos. (**b**) Immunostaining of GFP as surrogate for FADD and endothelial cell lineage marker PECAM on embryonic heart section of the indicated genotypes. Nuclei were stained by DAPI. (**c**) H&E staining on E11.5 embryonic section of the indicated genotypes
